# Progressive Medicine

**Published:** 1906-08

**Authors:** 


PROGRESSIVE MEDICINE, VOL. II, JUNE, 1906. A Quarterly Digest of Advances, Discoveries and Improvements in the Medical and Surgical Sciences. Edited by Hobart Amory Hare, M.D., Professor of Therapeutics and Materia Medica in the Jefferson Medical College of Philadelphia. Octavo, 368 pages, 31 illustrations. Per annum, in four cloth-bound volumes, $9.00; in paper binding, $6.00; carriage paid to any address. Lea Brothers & Co., Publishers, Philadelphia and New York.

This volume of Progressive Medicine begins with a valuable review of the recent progress in the treatment of Hernia, by Wm. B. Coly, M.D. Next comes Surgery of the Abdomen, exclusive of Hernia, by Edward Milton Foote, M.D. Ninety-four pages are devoted to this subject and includes all of the advances in this field of active work.

Nearly as much space is taken by John G. Clark, M.D., who reviews the year's work in Gynecology, and gives much concerning Carcinoma of the Uterus. Alfred Stengel, M.D., then follows with Blood Diseases of the Blood, Diathetic and Metabolic Diseases, Diseases of the Spleen, Typhoid Grand and Lymphatic System. Much time in recent years has been spent in this field of study, and Dr. Stengel has covered all the leading work. This volume concludes with "Ophthalmology," by Edward Jackson.

Like former issues of Progressive Medicine, the excellence of subject-matter and the manner in which it is handled deserve favorable commendation. We believe we are safe in saying that no other work succeeds in condensing so much scientific progress in available form as do the authors in each of these volumes.

